# Atlanta Society of Medicine

**Published:** 1890-02

**Authors:** 


### ATLANTA SOCIETY OF MEDICINE.

Dr. Cooper reported a case of fracture of the femur from a very unusual cause. The patient, a strong, well-developed boy of fourteen, was walking very rapidly on smooth, level ground; he stopped suddenly in his tracks, at the same time twisting his body half round to look at a boy who was following him; as he did so, he felt his left hip give way, and he fell helpless to the ground.

He was picked up and taken into the house, and the doctor summoned. After etherizing the patient, examination revealed a transverse fracture of the femur just below the trochanter, with marked shortening and great deformity. With the assistance of Drs. Harris and Jones the deformity was overcome, and a plaster of paris splint applied from the foot to the umbilicus.

The case is of interest because of the slight degree of violence which produced the fracture. In old persons fractures of the neck of the femur very commonly result from the application of trivial violence. This is entirely dependent on senile changes in the structure of the bone, and the inclination of the neck to the shaft.

In young people, however, it is very unusual to see a fracture of the femur frum such an insufficient cause. The history of the boy furnished a rational explanation of the accident: For the past three years he has suffered from pain in the hip, supposed to be rheumatic; this pain was not constant, being a great deal worse in winter than in summer. Frequently for a month or two at a time the pain was sufficient to make him walk with quite a perceptible limp.

It is very reasonable to believe that this pain was caused by a chronic inflammatory process in the upper end of the femur, possibly of a tubercular character. This slow inflammation resulted in osteo-porosis, a condition in which the interstitial spaces of the cancellous structure are increased in size, thereby weakening the bone. These condititions being present, a very slight violence would produce a fracture.

He also reported a case of amputation of the breast for malignant disease. The patient was a middle-aged lady, who had had for nearly thirty years an adenoma of the left breast, which caused her no inconvenience whatever. About six months ago the breast became the seat of severe neuralgic pains, and a gradual retraction of the nipple took place. The Doctor was first consulted last October. At that time the tumor was about the size of a small egg, hard and dense; the nipple much retracted, and the skin around it adherent and slightly ulcerated; no involvement of the axillary glands.

Immediate removal was urged, but the patient postponed operation until ten days ago, when, with the assistance of Drs. Elkin and Harris, he removed the breast. The highest temperature the patient had after the operation was 100 F.

This case illustrates the tendency of benign growths of the breast to undergo malignant transformation.

Dr. Nicolson reported two cases in which the subsequent history had been followed after operation for malignant growths. In one he had one year since amputated the thigh in the upper third for osteosarcoma of the femur, involving the bone as far as the middle. The patient made a rapid recovery, and was back at his home in a neighboring state on the twenty-first day from the time he left. The subsequent history was that he apparently did well for some time; the growth never returned in the bone, but after an illness covering a comparatively short time, he died a few days since from the secondary deposits in the lungs.

In the second case the patient was operated upon four years since for extensive epithelioma of the penis. There was such involvement of the lymphatic glands of both groins that it was only anticipated that the relief would be of a temporary nature. The operation was made with the Paquelin cautery, the penis being supported with an ivory staff introduced into the urethra, and the tissues burned through at a red heat. As an anæsthetic cocaine was successfully employed. There was no undue contraction of the urethral orifice, and the recovery was excellent. Recent report from him was to the effect that the glandular involvement had disappeared, and that he has been in perfect health, with no prospect of return. The remaining stump was pronounced eminently satisfactory and useful· About one inch of the organ was left.

Dr. Virgil O. Hardon reported a case of fibroid tumor of the ovary and exhibited the specimen. The patient, a white woman fifty-four years of age, ceased to menstruate three years ago. Ten months ago she first noticed a lump in the right groin, which gradually but steadily increased in size. About three months later the abdominal cavity began to fill with fluid, which rapidly increased until it distended the abdomen beyond the size of a pregnant uterus at term. Her general health failed, and she became extremely emaciated. Examination showed the abdomen to be filled with ascitec fluid. In the right inguinal region could be felt a tumor floating in the fluid and giving a very decided sensation of *ballottement* on deep pressure. It was freely movable, and apparently larger than a fœtal head. A diagnosis was made of solid tumor of the ovary complicated by ascites. January 20, 1890, laparotomy was performed. The ascitec fluid was evacuated through a small incision, and was estimated at three gallons in quantity. A solid tumor, twice the size of a fœtal head, was found lying in the right inguinal and lumbar region, and attached by a small pedicle to the right broad ligament. The incision was enlarged to five inches, the tumor removed, the pedicle ligated with silk and the abdomen sponged out and closed without drainage. The patient made a good recovery.

The tumor, which weighed three and a half pounds, was found to be a fibroid tumor of the right ovary. This variety of ovarian tumor is very rare. It is almost always accompanied by ascites, and is characterized by freedom from adhesions and a small pedicle consisted of the peritoneal investment of the ovary and the ovarian blood-vessels. After the removal of the tumor the ascites does not return. The causation is unknown.

In connection with the case of Dr. Hardon, the following remarks were made by Dr. J. McF. Gaston: The difficulty in diagnosticating an accumulation of fluid in a cyst from a collection in the peritoneal cavity in the form of ascites, when the abdomen was entirely filled, could not be satisfactorily solved by assuming that swelling of the feet and ankles accompanied ascites, as it was known to all who had observed cases of abdominal dropsy that extensive accumulation of fluid was frequently free from any dropsical effusion in the lower extremities. He did not consider the distinction based upon the lateral protrusion of the fluid upon median pressure of the abdomen as trustworthy, nor the distension of the false ribs by the accumulation, since a unilocular cyst had been observed on several occasions so completely filling the abdomen as to produce the same effect in those respects as an extensive ascites. In the case of adhesions of the sac to the periotis, the sense of fluctuation and other mechanical manifestations by palpation are identical in a large cyst and in ascites.

As to the recognition of the solid portion of an ovarian cyst, the mobility may be such as to lead to the same impression of a floating tumor as in this case, excepting the description of ballottement, which is only explained by the slender attachment of the mass.

He regarded the history of the formation of cystic tumors in a circumscribed portion of the abdomen as the only correct ground for distinguishing them from ascites.

Touching the report of the extirpation of the mammary tumor by Dr. Cooper, Dr. Gaston remarked that he had examined the case some weeks before the operation, and concurred in the diagnosis of its being a malignant growth. But he had since removed a tumor involving the mammary gland in a patient at Dawson, in which almost identical features of discoloration of the cutaneous covering, with retraction of the nipple and a sanious discharge, without enlargement of the axillary glands, had proved deceptive. The neoplasm proved, upon removal, to be a fibro-cystic tumor, with a dark, coffee-ground fluid, in one of the cysts, which, upon being evacuated accidentally in the dissection, caused apprehension on the part of one of the medical assistants that an aneurism had been opened. This gave no concern to the operator, as he was at once assured of its true character, and proceeded to remove the tumor with the entire mammary gland.

Another case had been operated upon in this city two weeks previously, which gave no signs of fluctuation, and turned out to be a fibro-cystic tumor of the breast, requiring the extirpation of the entire mammary gland. Neither of these cases presented any enlargement of the axillary glands; but he stated that another case had been examined something over a year ago, presenting a mass corresponding very much in its physical characteristics with the above cases, but with the additional feature of enlarged lymphatics in the axilla. A colleague had removed the tumor with the enlarged lymphatics, but the disease returned, and he had learned of the death of the patient a few weeks ago from the malignant tumor.

Dr. Gaston stated that the involvement of the axillary glands was a characteristic of the advanced stage of malignant tumors, and that it was rarely present in non-malignant tumors, though of long standing. It was therefore held to be an important element in the diagnosis of benign and malignant tumors to ascertain the existence or absence of enlarged lymphatic glands in the axillas of the affected side.

A case of impermeable stricture, with retention of urine, was reported to the Society by Dr. J. McF. Gaston. The patient had been originally under the care of Dr. Olmsted, who had withdrawn the urine by aspiration of the bladder prior to sending the man to the Providence Infirmary. Finding the bladder considerably distended again on the following day, Dr. Gaston made a puncture above the pubis at the same point with the aspirator needle, but without suction, and drew off the urine. He concluded that external urethotomy was called for, and invited Dr. Olmsted and Dr. Elkin to meet him next morning, when he extended the courtesy to Dr. Olmsted to complete what he had so judiciously begun in the aspiration. Incision being made in the media line between scrotum and anus, an abscess was evacuated, and the knife, guided by a sound, was carried along the urethra into the bladder. The incision was carried through the neck of the bladder to secure the involuntary discharge of the urine subsequently, as a means of relief to the cystitis, which was accompanied by thickening and indenture of the walls of the bladder, perceived distinctly by palpation, while there was likewise marked sensitiveness upon pressure.

An external application of an ointment of belladonna and mercury, each one-half ounce, pulv. camph. one drachm, iodine grs. v., iodide potash grs. vx., was made over the pubic region.

To correct septic manifestations, he took for some days sulf. quin. and muriate tinct. of iron.

There being subsequently indications of septic trouble, he was given calomel and Dov. powders, followed by cream of tarter, and the latter had been used since whenever a laxative was required.

The fluid ext. of buchu, with sweet spirits of nitre, has been given latterly, three times a day, with a good effect. The general condition of the patient being satisfactory, while the external wound has nearly closed, with a dripping of urine at times from the meatus, when the bladder is evacuated by voluntary effort. An operation is contemplated at an early day for division of the strictures of the urethra in its penile portion.

The existence of a suppurating sulcus along the urethra at the time of the external urethotomy, precluded a satisfactory result of an operation for opening the penile urethra on that occasion.

Dr. Elkin will be entrusted with the further management of the case, and the result will be given at a future meeting.

Dr. J. W. Duncan reported a case:

On January 18th (inst.) I was called to see a little girl, aged 7 years. She had been suffering very much during the day with a pain in right leg, below the knee, and was still suffering. There was no tenderness on pressure, or on motion. The pulse was 125, tongue coated and red at tip and edges. I diagnosed the case, from the general condition of the patient, to be something more than a simple neuralgic or rheumatic pain in the leg, and expressed the opinion that the trouble was in the abdomen. I gave her salol. grs. 2, camph. Dover's powder gr. 1, lactopeptine grs. 3, and calomel gr. 1-2 every three hours. She suffered all night with the pain in leg. Next morning the pain in the leg was gone, or nearly so, and she had well marked symptoms of enteritis. I used turpentine and lard on flannel cloths over abdomen, continued the powder, less the calomel, and gave her hydrarg. bichlor. gr. 1-64, tinc. opii deod. gtts. 4, aq. cinnam. ʒ 1-2, every three hours. 7 p. m. no better. Gave in addition an emulsion of castor oil, turpentine, etc., since which time she has gradually improved up to the present time (fifth day). The only peculiarity about the case was the location of the pain for the first 24 hours, after which the pain was confined to the small intestines, as is common in enteritis.

Dr. J. G. Earnest reported the following unique case:

In July last he, together with Dr. Ridley, was called by Dr. Goldsmith, of this city, to see a young lady in labor with her first child. She had been having active pains for thirty-six hours, and the membranes had ruptured several hours before his arrival. The head was found presenting at the superior strait—having made but little progress toward the outlet. Immediately in front of, and a little to the left of the well-dilated *os uteri*, could be felt through the coats of the vagina, a hard tumor the size of a small orange, bulging into the vagina and blocking the passage in such a way as to prevent the descent of the head. The uterus being firmly contracted, it was impossible to push the fœtus back into the womb, or in any way get it above the brim of the pelvis. Traction on the tumor with the fingers convinced him that the tumor could probably be brought down far enough to drop back into the space between the coccyx and the ischium. With this idea, the forceps were applied to the head, and after an hour of careful manipulation (the patient being under ether), the child was safely delivered. It was found, on examination, that the tumor had disappeared from the vagina. After the delivery of the afterbirth, the tumor was again searched for, and found lying loose in the abdomen, a little above and to the left of the contracted uterus. The mother and child did well until the sixth day after the delivery, when the patient developed a case of puerperal insanity. The case proving obstinate, it was thought best to place her in an asylum as soon as able to travel. After a few weeks' absence, she returned completely restored and in good physical condition. The tumor was found to be little changed from what it appeared at the time of the delivery. A careful examination convinced Dr. Earnest that it was of ovarian origin, and should be removed. Accordingly, he operated January 4th, 1890, assisted by Drs. McCaully and Huzza. The tumor proved to be a dermoid cyst of the left ovary, the size of a medium orange ; was hard as a base-ball, and when opened was found to contain a fleshy portion the size of an English walnut, in which was imbeded two well formed bicuspid teeth, and from which sprang a tuft of hair completely filling the cyst. It was saturated with the usual chyle-like fluid. The patient made a good recovery.

